# Early detection, care and control of hypertension and diabetes in South Africa: A community-based approach

**DOI:** 10.4102/phcfm.v12i1.2160

**Published:** 2020-02-20

**Authors:** Sanele Madela, Shamagonam James, Ronel Sewpaul, Siyathokoza Madela, Priscilla Reddy

**Affiliations:** 1 Expectra Health Solutions, Dundee, South Africa; 2 Private, KwaZulu-Natal, South Africa; 3 Social Aspects of Public Health, Human Sciences Research Council, Cape Town, South Africa

**Keywords:** community health workers, hypertension, diabetes, KwaZulu-Natal, non-communicable diseases

## Abstract

**Background:**

Non-communicable diseases (NCDs) are a major public health concern with raised blood pressure and glucose emerging as leading causes of death and disability.

**Aim:**

This community-based demonstration project using community caregivers (CCGs) trained in screening for hypertension and diabetes aimed at improving early detection and linkage to care and management.

**Setting:**

The project was conducted in KwaZulu-Natal province.

**Methods:**

The CCGs were trained in NCD-related health education, promotion and screening for hypertension and diabetes using an accredited programme. The CCGs screened community members for hypertension and diabetes using three screening methods: door-to-door visits, community campaigns and workplaces.

**Results:**

Twenty-five CCGs received the accredited NCD training. A total of 10 832 community members were screened for hypertension and 6481 had their blood glucose measured. Of those screened, 29.7% and 4.4%, respectively, had raised blood pressure (≥ 140/90 mmHg) and blood glucose (≥ 11.0 mmol/L) who required referral to a primary healthcare facility. More than one in five (21.0%, *n* = 1448), of those with no previous hypertension diagnosis, were found to have raised blood pressure at screening, representing newly detected cases. Less than a third (28.5%) of patients referred to the facilities for raised blood pressure actually presented themselves for a facility assessment, of which 71.8% had their hypertension diagnosis confirmed and were advised to continue, adjust or initiate treatment. Similarly, 29.1% of patients referred to the facilities for raised blood glucose presented themselves at the facility, of which 71.4% received a confirmatory diabetes diagnosis.

**Conclusion:**

Community caregivers played an important role in early detection of raised blood pressure and raised blood glucose, and in referring patients to primary care.

## Introduction

Globally, and particularly in sub-Saharan Africa, non-communicable diseases (NCDs) are emerging as a major public health concern. The Global Burden of Disease, Injuries and Risk Factors Study 2017 reported a 40% increase in early deaths and disabilities from 1990 to 2017 attributed to NCDs, with the leading risk factors for disability and death being raised blood pressure (BP), smoking and raised blood sugar.^1^ Furthermore, while global life expectancy was 73 years, healthy life expectancy (HALE) was 63 years – meaning, on average, 10 years was spent in poor health.^1^ Efforts to improve life expectancy need to be balanced with increasing or improving interventions that enhance HALE. Such interventions may address risk prevention and behaviour change as well as upstream determinants of health such as policies regarding living conditions and provision of health services.^2^ Factors such as rapid urbanisation, dietary changes and physical inactivity seem to be driving the NCD burden in low- and middle-income countries (LMICs).^3^

The NCD management in LMICs is characterised by a general lack of attention to health promotion at all levels of care. These include early detection, as well as care and management, to prevent disease progression and complications.^4^ Furthermore, the fragmented health system tends to lean towards addressing more visible communicable conditions like HIV and AIDS. In South Africa (SA), despite the slight decrease in deaths related to NCDs in 2010, NCDs still accounted for 38% of mortality, with a higher percentage for women (42.6%). Cardiovascular disease (CVD) and diabetes mellitus were among the top four NCDs resulting in death.^5^ More than a third (36%) of NCD-related deaths occurred before the age of 60 years.^6^

Models of NCD detection, care and management are needed to meet SA’s challenge. Worldwide, it is recognised that NCD management is an onerous task. It requires intervening at different levels from prevention, early detection to basic and complex care and management of patients over their lifespan.^7^ At community level, harnessing opportunities for prevention, screening, early detection, referral and linkage to care is needed to prevent and/or delay the onset of potential NCDs.

Restructuring the health services to include testing and evaluating new models of care designed to provide good-quality NCD care, based on policy and supported by governance structures for sustainability and scalability, is needed.^8^ One such model is the integrated chronic disease management (ICDM) model that is developed mainly from lessons learnt in the delivery of SA’s HIV and AIDS programme.^9^ The HIV and AIDS programme is distinguished by several novel strategies and activities that contribute to its comprehensive management of patients from a primary healthcare (PHC) perspective and a focus on continuity of care.^3,10^ Continuity of care requires several components, with one component being a specific cadre of healthcare providers. Healthcare providers are the front-line staff of PHC, which is considered to be the gateway to the health system for many patients.^3^ The ICDM model is a patient-centred model focussed on empowering the patient as well as integrating care at a community and health service level.

South Africa in its pledge to ‘improve the health of all’ has embarked on a clear strategic plan to revitalise the health system as outlined in its PHC re-engineering plan^11^ and ICDM manual.^9^ These strategic documents provide guidance for re-orienting the health system from the focus of infectious diseases, in particular HIV and AIDS, to addressing NCDs from a PHC perspective. Implementing strategies to intervene in an early and timely manner is imperative to reducing complications like strokes and heart attacks associated with NCDs. The feasibility of interventions being implemented successfully depends on a core set of delivery prerequisites, such as adequately trained personnel at relevant points in the healthcare spectrum.^8^

The ICDM model^9^ consists of four inter-related phases, one of which is the development of ward-based outreach teams (WBOTs). The role of the WBOTs is to empower individuals to take responsibility for managing their own conditions and increasing awareness of chronic diseases at the population level. This role is currently being conducted by Department of Health community caregivers (DoH CCGs) whose main function is to provide HIV home-based care and management, leaving a distinct gap around the identification, linkage to care and management of NCDs at community level. Based on this, CCGs are a key health force upon which to focus by equipping them with NCD skills training (including obtaining clinical measures), thereby creating an enabling environment for community-based healthcare delivery and improved liaison with PHC systems.^12^

The goal of this article is to describe the implementation of a community-based programme, namely, HealthRise South Africa (SA), which equipped a cadre of healthcare workers – CCGs – to perform tasks such as screening and clinical measures in the general population. This initiative aimed to enable early detection of risk factors for NCDs and promote referral and linkage to care as well as promote the management of patients having NCDs such as hypertension and diabetes. The article presents process evaluation results of the HealthRise SA programme in uMgungundlovu district municipality in KwaZulu-Natal (KZN) to demonstrate the programme implementation and reach. Specifically, it seeks to demonstrate the extent to which NCD-trained CCGs were able to conduct screenings for hypertension and diabetes, identify individual risks of community members and refer them to a local healthcare facility for confirmatory diagnosis and treatment.

## Methods

### Study design

**FIGURE 1:**
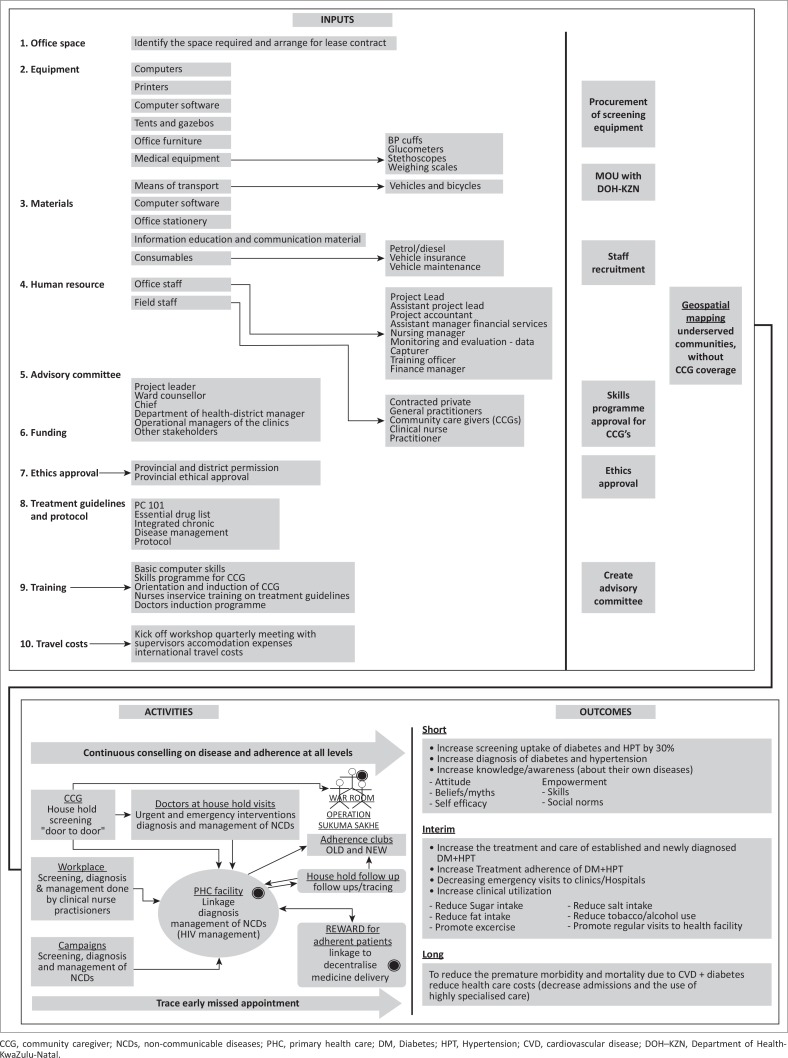
Logic model for the HealthRise South Africa project in uMgungundlovu district.

HealthRise SA was established as a community-based demonstration project by Medtronic Foundation, a global philanthropy, after negotiations with the Provincial and National Departments of Health. The logic model for the HealthRise SA programme in uMgungundlovu district is shown in Figure 1. Participants aged ≥ 15 years were accessed from the communities using three methods: door-to-door household visits, community campaigns and workplace visits. A process evaluation was conducted, where outputs including numbers screened, referred and diagnosed were used to assess programme implementation in the screening and referral phases of the programme.

### Setting

HealthRise was established as a demonstration project designed to improve screening, diagnosis, management and control of hypertension and diabetes in underserved communities. The other countries that participated in the global HealthRise initiative were Brazil, India and the United States. Implementing organisations in each location developed programmes tailored uniquely to each local context and a common feature of the programme was empowering front-line health workers.^13^ HealthRise SA was conducted from February 2017 to February 2018 in two independently implemented project sites, one in Pixley Ka Seme district in the Northern Cape province and the other in the uMgungundlovu district in KZN, the latter of which is the focus of this article.

Expectra Health Solutions (EHS), a local non-profit organisation, was commissioned to conduct this demonstration project in uMgungundlovu district municipality. This district is 1 of 11 in the province. It was chosen because it is one of the National Health Insurance (NHI) pilot sites, and characteristic features of the pilot sites include being an underserved area with poor health outcomes and a health system in need of improvement.^14^ Furthermore, uMgungundlovu is a district with a high burden of NCDs, with 46.8% and 11.0% of the population being hypertensive and diabetic, respectively.^15^ These rates were comparable to the rest of the country.^16^ The incidence of hypertension in KZN in 2016 and 2017, expressed as the number of newly diagnosed cases per annum per 1000 population aged 40 years and older, was 21.7, which was higher than the national incidence of 18.9. Among the KZN districts, uMgungundlovu had the second highest hypertension incidence at 24.4. Similarly, KZN’s diabetes incidence rate of 2.8 newly diagnosed cases per 1000 population aged 40+ years was higher than the national incidence of 2.5. uMgungundlovu district had the highest diabetes incidence in the country at 8.3.^17^ uMgungundlovu has a mobile population where a large proportion of people live in the urban areas for work purposes.

HealthRise SA was conducted in the Msunduzi, uMshwathi and Mkhambathini subdistricts of uMgungundlovu.

### Training of community caregivers

For this demonstration project, 25 people were recruited from the community to be trained as CCGs (EHS CCGs). They were recruited using set criteria, namely, communication skills, minimum of matric education, previous work experience in healthcare or as a volunteer CCG for > 1 year and a passion for helping the communities in which they reside.

A 3-day NCD skills training programme was developed for these CCGs. It was based on an accredited community health programme developed by EHS and approved by the Health and Welfare Sector Education Authority (HWSETA). All training adhered to the DoH protocol and guidelines including Adult Primary Care, ICDM, Diabetes Type 2 Management Guidelines and the Ideal Clinic concept, with view to possible scalability of the programme.^18,19^ The training was facilitated by an EHS facilitator, who was a medical doctor with training in a public health approach. The content included communication skills, NCD-related health education, health promotion, measuring heights, weights and waist circumference and screening for hypertension and diabetes, using standardised procedures for BP and blood glucose measurement. The CCGs were trained to use glucometers and digital BP machines through a process of demonstration and practice until competence was reached. A second layer of training included establishing competence in administering the patient questionnaires, and tracking and administrative procedures. The 25 participants had to complete a practical assessment of taking BP, blood glucose and anthropometric measurements as well as to demonstrate knowledge and application of the programme content. All 25 participants displayed adequate skills and knowledge in their assessments and were employed to work as CCGs on the project. During the implementation, the conduct of their tasks was supported and there was ongoing oversight.

From the outset, the project was implemented in full consultation and participation of the KZN DoH. It was therefore crucial to build the capacity of the DoH staff in NCD work. This required training of the DoH CCGs to match the level of skill of the EHS CCGs. Furthermore, clinical nurse practitioners and professional nurses from the facilities were trained to increase their knowledge about NCDs and garner their support for the management and care of NCD patients referred to their facilities.

### Study population and sampling strategy

A cross-sectional approach was used for the initial phase of this demonstration project. Seven PHC facilities were selected from across the Msunduzi, uMshwathi and Mkhambathini subdistricts. These were purposively selected using predefined criteria in a consultative process with the KZN DoH. The criteria included facilities with no or minimal DoH CCGs. The selected facilities were also required to represent a combined catchment area that comprised residents living in rural informal, rural formal (farm), urban formal and urban informal areas. The seven facilities had a catchment population totalling 163 995 people.

All individuals aged ≥ 15 years were eligible for screening. They were approached using three methods, namely, door-to-door home-based visits, campaigns and workplace visits. Using the DoH guidelines for CCGs, it was determined that each pair of CCGs could access an average of 15 households per day, which over the 13-month period would result in screening a targeted number of over 10 500 people.

Households and workplaces that were approximately within a 5 km – 10 km radius of each facility were visited. Door-to-door visits were conducted where a CCG started from one end of each street and visited every household up to the other end of the street during working hours. However, within the catchment areas, EHS CCGs would not work in areas where DoH CCGs (with their mainly HIV mandate at the time) were already working. This was to avoid interfering with the DoH CCG work and causing possible confusion of multiple CCGs visiting community members on the same day.

Once the home was accessed every eligible and consenting household member was screened for BP and blood glucose. Overall 10 658 households were visited with an available population of 11 860 household members. Of these 7954 people consented to be screened. Sixteen workplaces were visited comprising farms and factories, from which 525 people were screened. Screenings were conducted at workplaces to accommodate employed community members who were not available at home or were unable to visit a health facility because of work commitments. Twenty-four campaigns were conducted at community centres from which 2353 people were screened. A total of 10 832 people were screened by HealthRise SA KZN.

### Data collection

**FIGURE 2:**
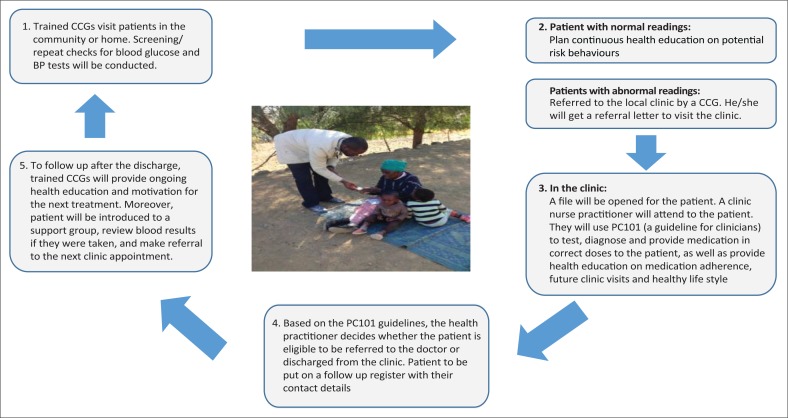
A flow chart of a patient-centred care model for the Expectra Health Solutions HealthRise project.

Using a patient-centred approach, data on demographics and socio-economic status of all family members were collected. They were then screened for hypertension and diabetes, which was followed by a facility referral, where necessary (Figure 2). The instruments used included the family profile questionnaire and an individual screening questionnaire. The questionnaires were pre-tested among a small convenience sample of community members and researchers for comprehensiveness and the instruments were refined accordingly.

The family profile questionnaire was used to obtain data on all family members, including demographics, highest level of education, employment, specific questions for women on pregnancy and breastfeeding, type of birth and immunisation status of children in the family, sources of income and grant status. The family profile was used to assess which family members were aged ≥ 15 years and were therefore eligible to be screened.

The individual-level general screening questionnaire was administered to individuals aged ≥ 15 years. It included items on risk behaviours, demographics, diabetes risk assessment, previous diagnoses of hypertension and diabetes, current medication use and previous diagnoses of communicable conditions and other NCDs. Waist circumference, heights and weights were obtained from consenting individuals.

Measurement of BP and random blood glucose was conducted using standardised techniques prescribed in the South African Department of Health’s Adult Primary Care guidelines.^19^ Blood pressure was obtained using digital BP machines. A range of different BP cuff sizes was available for use depending on the patient’s arm size.

Individuals who had a BP reading ≥ 140/90 mmHg had their BP measured again 6 h later, or on the following day. If they were found to have a persistent BP of ≥ 140/90 mmHg, they were referred to the facility for a confirmatory diagnosis and possible initiation of treatment. Patients who were currently on hypertension treatment and who were found to have readings ≥ 140/90 mmHg were referred to the facility for adjustment of their treatment. Patients with a reading of ≥ 160/100 mmHg, whether newly detected or previously diagnosed, were immediately referred to the facility.

A blood glucose test was conducted on all persons aged ≥ 45 years. For individuals < 45 years old, the FINDRISC diabetes risk assessment^20^ was performed, which reported waist circumference, BP, family history of diabetes, tuberculosis (TB) status in the last year and gestational diabetes or a having had a baby weighing > 4 kg at birth. A blood glucose test was conducted for anyone with two or more risk factors. Blood was obtained via a finger prick. Individuals who had a random blood glucose level ≥ 11 mmol/L were immediately referred to the facility. Patients with a previous diagnosis and who had raised blood glucose ≥ 11.0 mmol/L were referred to the facility for adjustment of treatment. All data collected were captured by EHS CCGs into a registered mobile data collection platform using electronic mobile devices.

Community caregivers followed up patients who were referred to the facility after 1 week of their referral. If they did not visit the facility, reasons for not attending were assessed and a referral made to the nursing manager by telephone.

Monthly exports of the data collected were cleaned and analysed and used to calculate indicators on screening, referral, referral completion and diagnosis. The indicators were used to monitor programme performance and make necessary process adjustments along the 13 months of the programme.

### Data analysis

Data were analysed using Stata 14.0 (StataCorp, TX, USA).^21^ Process indicators on screening, referral, completed referrals and diagnosis were calculated by screening method. Descriptive statistics were calculated that reflect the health and socio-demographic characteristics of the sample and the prevalence of detected cases of raised BP and blood glucose.

### Ethical considerations

The study was conducted in accordance with the International Ethical Practice for Research with Human Subjects. Written informed consent was obtained from all participants. Participants who were unable to read and/or write had the consent forms read out to them and a finger print was obtained. Ethical approval for the study was obtained from the Human Sciences Research Council of South Africa (HSRC) Research Ethics Committee (REC). Protocol Number: REC 4/21/09/16. The HSRC REC is registered with the South African National Health Research Ethics Council (REC-290808-015). The HSRC REC has US Office for Human Research Protections (OHRP) Federal-wide Assurance (FWA Organisation No. 0000 6347).

## Results

### Demographic and socio-economic profile by the three screening methods

Of the 10 832 individuals screened, 51.2% were 31–60 years old and 26.3% were older than 60 years (Table 1). Almost three-quarters (71.9%) were women and 93.5% were of African ethnicity. When compared with the demographic distribution of uMgungundlovu district,^22^ the screened sample had a higher female-to-male ratio and a higher proportion of people older than 60 years.

**TABLE 1:** Description of the sample and the screening outcomes by screening platform.

Characteristic	Total screened		Household visits		Community campaigns		Workplace visits		*p* for linearity
	%	freq.	%	freq.	%	freq.	%	freq.	
**Sex**									0.002
Male	28.1	2817	28.0	2044	26.9	591	34.7	182	
Female	71.9	7209	72.0	5257	73.1	1609	65.3	343	
**Age group (years)**									< 0.001
Mean age (s.d.)	47.2	17.8	46.3	17.6	51.5	18.8	41.3	13.2	< 0.001
15–17 years	1.0	113	0.9	75	1.5	36	0.4	2	
18–30 years	21.5	2325	22.5	1793	17.4	410	23.2	122	
31–60 years	51.2	5549	52.8	4203	41.7	982	69.3	364	
over 60 years	26.3	2845	23.7	1883	39.3	925	7.0	37	
**Ethnicity**									< 0.001
African	93.4	10 122	94.0	7473	91.7	2157	93.7	492	
Mixed race	1.8	193	1.7	132	2.5	59	0.4	2	
Indian	4.4	476	3.9	312	5.8	136	5.3	28	
Other	0.3	37	0.4	33	0.0	1	0.6	3	
White	0.0	4	0.1	4	0.0	0	0.0	0	
**Previous diagnosis of hypertension**									< 0.001
No previous diagnosis	63.5	6882	67.2	5344	51.1	1202	64.0	336	
Previously diagnosed and not on treatment	9.0	973	5.3	423	18.3	432	22.5	118	
Previously diagnosed and on treatment	27.5	2977	27.5	2187	30.6	719	13.5	71	
**Previous diagnosis of diabetes**									< 0.001
No previous diagnosis	80.5	8725	85.7	6814	65.3	1537	71.2	374	
Previously diagnosed and not on treatment	8.5	926	3.4	272	22.5	530	23.6	124	
Previously diagnosed and on treatment	10.9	1181	10.9	868	12.2	286	5.1	27	
**Hypertension screening outcomes**									< 0.001
Normal blood pressure	70.3	7618	73.1	5813	60.4	1420	73.3	385	
Raised blood pressure (≥ 140/90 mmHg)	29.7	3214	26.9	2141	39.7	933	26.7	140	
**Diabetes screening outcomes**									< 0.001
Not eligible for screening	40.2	4351	41.6	3305	33.4	785	49.7	261	
Normal blood glucose	55.4	6002	54.4	4323	60.6	1426	48.2	253	
Raised blood glucose (≥ 11.0 mmol/L)	4.4	479	4.1	326	6.0	142	2.1	11	
**Total**	**100.0**	**10 832**	**100.0**	**7954**	**100.0**	**2353**	**100.0**	**525**	**-**

s.d., standard deviation.

More than a third (36.5%, *n* = 3950) reported that they had previously been diagnosed with hypertension. Of those with previous hypertension diagnoses, three-quarters (75%, *n* = 2977) reported currently taking treatment. Almost a fifth (19.5%, *n* = 2107) of the screened sample reported that they had previously been diagnosed with diabetes; 56% (*n* = 1181) of whom were currently taking diabetic treatment.

The majority of screened individuals (73%, *n* = 7954) were obtained from household visits, 21.7% (*n* = 2353) at community campaigns and 4.8% (*n* = 525) at workplace visits. Sex, ethnicity and age group distributions differed between the three screening methods. There was a higher proportion of men screened at workplaces than at community campaigns and households. The mean age of individuals screened at community campaigns was 51.5 years, which was significantly higher than those screened at households (46.3 years) and workplaces (41.3 years). The proportion of individuals with previously known hypertension and diabetes diagnoses was lowest among those screened at households, while community campaigns yielded the highest proportions of individuals with known previous diagnoses.

### Identification of hypertension and diabetes status

Of the 10 832 screened individuals, 29.7% were found to have raised BP of ≥ 140/90 mmHg. With respect to screening for diabetes, 40.2% were not eligible for diabetes screening (they were < 45 years old or had no or minimal risk factors for diabetes according to the diabetes risk assessment); 55.4% had normal levels of random blood glucose and 4.4% had a raised blood glucose of ≥ 11.0 mmol/L who required referral to a facility for confirmatory diagnosis.

A higher proportion of individuals with raised BP was found among those screened at community campaigns (39.7%) than at workplaces (26.7%) and households (26.9%). Community campaigns also yielded the highest proportion of individuals with raised blood glucose (6.0%), followed by household screenings (4.1%) and workplace screenings (2.1%).

Of the 6882 individuals without a prior hypertension diagnosis, more than one in five (21.0%) was found to have raised BP, thus representing newly detected cases of hypertension (Table 2). Similarly, of the 8725 individuals without a prior diabetes diagnosis, 1.4% were newly detected cases of raised blood glucose.

**TABLE 2:** Raised blood pressure and blood glucose at screening among those with and without a previous diagnosis of their condition.

Knowledge of previous diagnosis of the condition	Hypertension			Diabetes		
	Screened and found to have raised blood pressure ≥ 140/90 mmHg			Screened and found to have raised blood glucose ≥ 11.0 mmol/L		
	%	frequency	*N*	%	frequency	*N*
Not previously diagnosed	21.0	1448	6882	1.4	125	8725
Previously diagnosed and not on treatment	29.8	290	973	2.3	21	926
Previously diagnosed and taking treatment	49.6	1476	2977	28.2	333	1181
**Total**	**29.7**	**3214**	**10 832**	**4.4**	**479**	**10 832**

### Management of hypertension and diabetes

For those patients previously diagnosed and on treatment for hypertension, almost half (49.6%, *n* = 1476) had a raised BP at screening (Table 2). For those patients previously diagnosed with diabetes and on treatment, 28.2% (*n* = 333) had a raised random blood glucose level (≥ 11 mmol/L).

### Referral and linkage to primary healthcare

**FIGURE 3:**
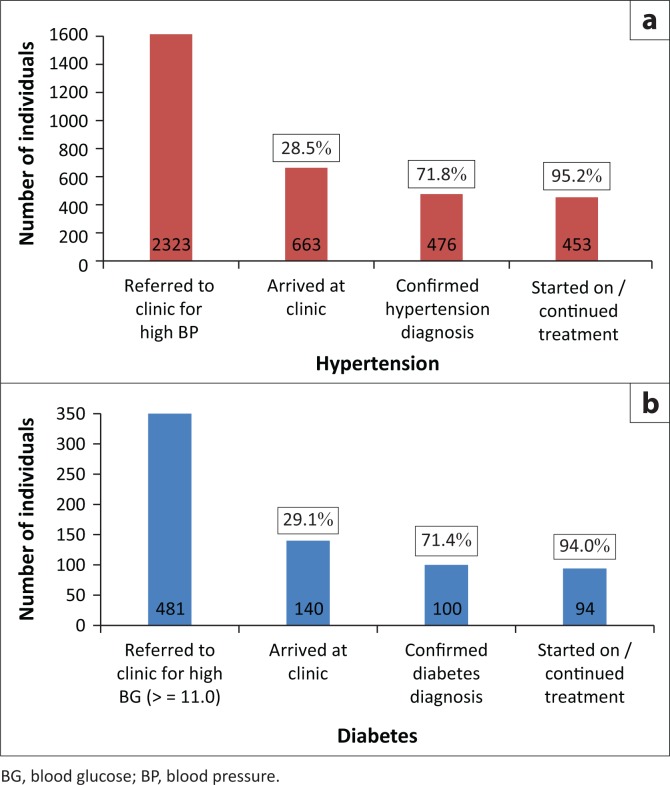
Referral, diagnosis and treatment initiation for hypertension and diabetes.

Figure 3 shows the referral, diagnosis and treatment initiation of the individuals who were screened and referred for hypertension and diabetes. Of the 2323 participants referred to the facilities for raised BP, only 663 (28.5%) arrived at the facility, 476 (71.8%) of whom had their hypertension diagnosis confirmed and 453 (95.2%) started or continued their treatment. Of the 481 participants referred to facilities for raised blood glucose, 140 (29.1%) arrived at the facility, 100 (71.4%) of whom were found to have a confirmed diabetes diagnosis and 94 (94.0%) were started onto or continued treatment.

## Discussion

This article explored the feasibility of trained CCGs to engage with communities and assess their hypertension and diabetes status. Existing PHC services are geared towards in-facility patient healthcare seeking for acute ailments. The tasks of CCGs are geared towards home-based care for patients with HIV and/or TB.

In this study, CCGs were trained to engage with communities, access homes, conduct campaigns, visit workplaces and obtain clinical measurements of BP and blood glucose. The findings reveal substantial numbers of participants with an unmet need for hypertension and diabetes education, care and management, as well as poor control and follow-up care of those previously diagnosed. For those participants who were referred and attended the facility, more than nine in 10 people started or continued with treatment.

This demonstration project draws attention to the substantial number of patients who did not visit the facility upon referral. Similar rates of following up at facilities after referral were found in another study with community health workers screened for CVD.^23^ This is cause for concern although it is not uncommon for patients to delay seeking care and seek care outside of their area of residence or from alternate or mixed sources of healthcare (herbalists and traditional healers).^7^ However, more research is needed to determine the actual reasons for the obstacles related to the continuity of care and to understand patients’ needs and preferences. Establishing a CCG workforce may help overcome some barriers by strengthening collaboration between facility and community-level staff. A feedback and referral system can be effectively used to enhance continuity of care.

In addition, NCD-trained CCGs may be able to provide on the spot-informed care to patients and help with educational efforts and management issues that may arise once a patient has left the facility following diagnosis. A South African study reported lowered blood glucose levels at 18 months following an empowerment education programme in the first 3 months of care.^24^ Community caregivers are also able to take on a health education role for prevention of risk factors by imparting awareness, knowledge and skills to those not affected by an NCD.^3^

It may be argued that this active form of case finding for NCDs is labour-intensive and the actual yield of linkage to care poor,^3^ the latter of which was confirmed in this demonstration project. However, active case finding for NCD risk in this project was a component of a more comprehensive PHC approach. Other elements like overall benefit to the community, and health seeking and uptake behaviours in the longer term, need to be assessed. Reliance on a passive case finding approach perpetuates the risk of late detection of NCDs with consequent complications and added burden of disease. Many NCD risk factors, such as overweight and obesity, are preventable and can often be addressed at the home and community level.^4^ Increasing the visibility and credibility of prevention services in the community will in the longer term assist prevention and management of NCD efforts.

In the South African context, a strong foundation has been laid for the management of HIV and AIDS using a PHC approach. A major focus will be to move from an infectious disease-specific focus to a focus on patients with a chronic condition or prevention of potentially chronic conditions – aimed at generic education on lifestyle and well-being.^25^ Such efforts will require commitment to modifying health systems at all levels, openness to innovative programmatic and service delivery. Moreover, role definitions and professional training may need to be modified to match changing healthcare needs.

The limitation of this project is that it was conducted in one district with characteristics such as high unemployment, low resourced health systems and high diabetes and hypertension prevalence, and therefore the findings may not be generalised to other districts in the province. The population screened was largely a mobile population residing in the city for purposes of work and this may have hampered follow-up visits. In rural areas, the long distances from the facility and possible financial constraints may have limited access to health services.

## Conclusion

This demonstration project draws attention to the unmet healthcare of people living with NCDs in communities. It also draws attention to the potential value of well-trained CCGs to bridge the gap in resource-limited communities by shifting work from specialist level down to community care level. More specifically, it calls for additional efforts focussing on referral of patients at risk following detection. Methods to motivate patients and facilitate their referral uptake are required. This calls for a systematic approach to NCD identification and linkage to care at community level using existing services nuanced by innovations that lend themselves to enhancing measures for early detection, scale-up and sustainability.
